# School Medical Inspection in the Transvaal

**Published:** 1916-06-17

**Authors:** 


### SCHOOL MEDICAL INSPECTION IN THE TRANSVAAL.

### A Record of Progress.

The second report of the Medical Inspector of Schools of the Transvaal is as interesting a document as its predecessor, an account of which has already appeared in The Hospital (November 19, 1915; p. 145). Like the previous report, this one does not deal with a full year's work, for the reason that the Inspector was seconded during some four or five months out of the twelve for service in the Defence Department. In spite of this the Medical Inspector has been able to complete the organisation of his department: his staff now consists of an assistant medical inspector (a medical woman, who takes over the work of inspecting girls' schools); two part-time medical inspectors, one of whom is an alienist and deals especially with mentally deficient children; an ophthalmologist for the thickly populated Witwatersrand area; and two school nurses. Two school clinics have been established as a commencement, and the nucleus of a treatment centre has been started. Unfortunately the equipment ordered from England for the latter had not arrived at the date of the report. The two clinics are at Pretoria and Johannesburg respectively, and are both by now in working order.

### School Planning.

In spite of the time which routine visiting requires in a community so large and scattered as the Transvaal, and of the demands of defence and of the organising of a new department, the Medical Inspector, Mr. C. L. Leipoldt, has managed to envisage very thoroughly the less obvious duties of his office, as well as those of a more commonplace kind. Thus he has prevailed upon the Public Works Department to submit to him all plans of new school buildings and of modifications of old ones; and the correct principles of school construction receive due attention in the report. While approving, in the main, the existing type of plans upon which the P.W.D. works as suitable for the more temperate districts of the province, he is in favour for small country schools in the northern and eastern districts (where the climate is much hotter) of a different type exemplified by a particular school in the Zoutpansberg. One of the outstanding features of this is a thatched roof, a feature which is not very rare architecturally in Cape Colony, but was unknown to the present reviewer as applied to the interior provinces.

The winter of 1915 (May to July) was an exceptionally severe one in the Transvaal; and the Inspector calls attention to the inadequacy of the heating arrangements in the High Veld schools. Lavatory accommodation and management are reported as in the main satisfactory; possibly the strictures on this subject in the last report have borne good fruit.

It says much for the Inspector's tact that but little trouble has been experienced with the parents over medical inspection of their children; probably no English inspector could have overcome so successfully the prejudices of the Boer agricultural classes as Mr. Leipoldt has evidently done. It was, further, a happy thought on his part to contribute a series of articles explaining the objects and methods of school inspection to a leading Dutch newspaper. By a piece of careless drafting in the Act which sought to establish medical inspection of schools the whole intent of the Legislature was nullified; and the curious position arises that there is no proper legal authority for the work of the Inspector. Some parents are acute enough to know this, and there is undoubtedly force in the plea for specific authority such as the Legislature intended, but failed, to give.

### The Hygiene of the Veld.

The statistics concerning cleanliness, prevalence of vermin, and personal hygiene in general, though incomplete, show improvement on those of last year. The Inspector discusses the depths of both parental and filial ignorance concerning the simplest rules of hygiene, and suggests that the compilation of an elementary text-book might usefully occupy his department. The special climatic conditions of South Africa render books dealing with these subjects from the point of view of Great Britain quite useless. The statistics of defective health show an apparent improvement; but the Inspector explains that he has had to content himself with a lower standard of what constitutes a child free from defects: that is, he has had to overlook a number of the slighter defects and mark those children as normal. This has been forced upon him by the unwillingness or inability of parents to have slight defects (teeth, tonsils, and so forth) properly treated, and is not adopted on grounds of principle but merely on those of expediency.

### The Ætiology of Rickets.

In dealing with specific defects the Inspector has some interesting remarks on the subject of rickets. This disease is exceedingly rare amongst the Transvaal school children, notwithstanding that many of the poorer classes in the country districts commit exactly those dietary errors in the feeding of their children to which the disease in Europe has been commonly attributed; namely, excess of carbohydrate foods with deficiency of fats. The Inspector believes he can identify in the Transvaal a clinical entity which results from this particular error of diet: but it is not rickets, and he suggests that these observations support perhaps the microbic theory of rickets which has lately been put forward. In certain districts bilharziasis is very prevalent amongst the children, and accounts for a good deal of impaired health and efficiency. The Inspector considers that Leiper's hypothesis as to the transmission of this disease by water snails is not yet proved. Tapeworm is another parasite which is much more troublesome in South Africa than it is in this country. Altogether the progress made is distinctly encouraging, and the special problems of a sub-tropical province at a high altitude are evidently being tackled with energy and judgment.

